# Cost-effectiveness of a vocational enablement protocol for employees with hearing impairment; design of a randomized controlled trial

**DOI:** 10.1186/1471-2458-12-151

**Published:** 2012-03-01

**Authors:** Arjenne HM Gussenhoven, Johannes R Anema, S Theo Goverts, Judith E Bosmans, Joost M Festen, Sophia E Kramer

**Affiliations:** 1 Department ENT/Audiology and EMGO Institute for Health and Care Research, VU University Medical Center, Amsterdam, The Netherlands; 2 Department of Public and Occupational Health and EMGO Institute for Health and Care Research, VU University Medical Center, Amsterdam, The Netherlands; 3 Research Center for Insurance Medicine AMC-UWV-VUmc, Amsterdam, The Netherlands; 4 Department of Health Sciences and EMGO Institute for Health and Care Research, Faculty of Earth and Life Sciences, VU University, Amsterdam, The Netherlands

**Keywords:** Hearing loss, 'Need for recovery after work', Economic evaluation, Psychosocial problems, Occupational physician, Integrated care, Intervention

## Abstract

**Background:**

Hearing impairment at the workplace, and the resulting psychosocial problems are a major health problem with substantial costs for employees, companies, and society. Therefore, it is important to develop interventions to support hearing impaired employees. The objective of this article is to describe the design of a randomized controlled trial evaluating the (cost-) effectiveness of a Vocational Enablement Protocol (VEP) compared with usual care.

**Methods/Design:**

Participants will be selected with the 'Hearing and Distress Screener'. The study population will consist of 160 hearing impaired employees. The VEP intervention group will be compared with usual care. The VEP integrated care programme consists of a multidisciplinary assessment of auditory function, work demands, and personal characteristics. The goal of the intervention is to facilitate participation in work. The primary outcome measure of the study is 'need for recovery after work'. Secondary outcome measures are coping with hearing impairment, distress, self-efficacy, psychosocial workload, job control, general health status, sick leave, work productivity, and health care use. Outcome measures will be assessed by questionnaires at baseline, and 3, 6, 9, and 12 months after baseline. The economic evaluation will be performed from both a societal and a company perspective. A process evaluation will also be performed.

**Discussion:**

Interventions addressing occupational difficulties of hearing impaired employees are rare but highly needed. If the VEP integrated care programme proves to be (cost-) effective, the intervention can have an impact on the well-being of hearing impaired employees, and thereby, on the costs for the company as well for the society.

**Trial registration:**

Netherlands Trial Register (NTR): NTR2782

## Background

Hearing impairment is a common chronic condition in The Netherlands and worldwide. Although the vast majority of adults with hearing impairment has already retired [1], there is a considerable number of younger persons with hearing impairment still active in the labor force. The prevalence of hearing impairment in the Dutch labor force is about 3% (255.000 people) [2]. These numbers are comparable with estimations of the global burden of hearing loss according to the World Health Organization (WHO) [3]. It is well established that hearing impairment is age-related [3]. The expectation is that the number of employees with hearing impairment will increase in the near future because of the ageing population, which have a higher prevalence of hearing impairment, together with plans to increase the retirement age. A problem for hearing impaired employees is that occupations nowadays more and more depend on communication skills. As described by Ruben [4], the modern western economics underwent fundamental changes during the second half of the 20th century. While society was largely dependent on manual labor in the past, occupations today rely more on communication abilities. This implies a greater burden on people suffering from hearing impairment. Altogether, this may lead to an increasing number of hearing impaired employees who need help for problems in the workplace situation.

So far, only a few studies have addressed the impact of hearing impairment on work performance. The studies that have been revealed that there is a range of concerns and problems that hearing impaired employees are likely to experience. Most frequently reported issues are lack of control, problematic functioning in environmental noise, concerns about job safety, sick leave, and the fear to loose employment [5-8]. Furthermore, fatigue and distress are considered as effects of hearing impairment arising due to the constant need to adapt to, and to compensate for the hearing impairment [9]. 'Need for recover after work' refers to the degree to which employees are able to recover from fatigue and distress after one day of work [10]. It is an important factor influencing an individual's physical and mental functional status, and thus the ability to cope with work. The model of Job Demand Control (JDC) makes a distinction between different psychosocial work conditions, such as psychological job demands and job control [11]. According to this model need for recovery can be seen as a short term effect of work [10]. A study by Nachtegaal et al. [12] showed significantly higher levels of 'need for recovery after work' among hearing impaired employees (46%), compared to normally hearing colleagues (38%). They also found that for every dB signal-to-noise ratio (dB SNR) poorer hearing test score, the need for recovery after work increased with 1.4 points. Due to these issues, hearing impaired employees are likely to experience problems varying from direct auditory dysfunctioning to more secondary issues such as psychosocial problems.

Hearing impairment has consequences not only for the working hearing impaired individuals themselves, but also for companies and society. Previous studies showed that hearing impaired people are overrepresented in the group taking sick leave from work [13] and that these persons received more health care [14] in comparison with normal hearing colleagues. This results in higher costs for both the company and society. Additionally, Mohr et al. [15] showed that most of the societal costs of hearing impairment are caused by reduced working productivity due to presenteeism (i.e., being present at work but not functioning at full capability).

A search of the literature showed that interventions specifically supporting hearing impaired employees have scarcely been described. Programmes, other than those aimed at preventing noise-induced hearing loss, for example the protocol of the Dutch Board for Occupational Medicine (NVAB) [16], are practically non-existent. It seems that in current audiological practices, the specific needs of hearing impaired people with work-related difficulties are not dealt with in a standard approach. In The Netherlands, usual care to handle with hearing impairment at work consists of the common guidance and (workplace) advices from the occupational physician (OP). The OP can refer the employee to the general practitioner, to the ear nose and throat (ENT) specialist, or to an audiologic clinic for further audiological diagnostics and care [17]. The possible hearing aid fitting is done by a hearing aid dispenser. There is anecdotal evidence indicating that this usual care does not solve all the problems at the workplace that arise from hearing impairment [8]. It is suggested that a timely coordination of multidisciplinary services is required to fully support and facilitate employees with hearing impairment [8,9]. Additionally, it is argued that if not taken care adequately, disabilities in the workplace due to hearing impairment may result in a substantial health problem and productivity loss with substantial costs for the society as a whole. Therefore, interventions in aural rehabilitation specifically focusing on work-related problems for hearing impaired employees are needed to reduce costs.

An integrated care programme to facilitate and improve the working situation for hearing impaired employees was developed by Kramer et al. [18]. The International Classification of Functioning, disability and health (ICF) provided the theoretical framework for this integrated care programme [19]. In this framework, not only the individual's health condition (hearing impairment at the workplace) but also environmental and personal factors play an important role. A hearing impaired person's disability and functioning should be seen as outcomes of interactions between an individual's health condition, activities, participation, environmental factors (room acoustics, detection of sounds, colleagues, task schedules), and personal factors (age, cognitive capacities, coping styles, education). The integrated care programme for hearing impaired employees, the Vocational Enablement Protocol (VEP), addresses all these factors in the ICF model to improve the participation of hearing impaired employees. The VEP integrated care programme consists of a multidisciplinary assessment of auditory function (body structures and functioning), personal characteristics (personal factors), workplace characteristics (environmental factors), and work demands (activities, participation). The main aim of the VEP integrated care programme is to facilitate participation in and retention of work for hearing impaired employees.

Satisfaction and experiences with the VEP integrated care programme is already evaluated and described elsewhere [18]. However, it is not yet known whether this integrated care programme is effective and cost-effective in comparison with usual care. Randomized controlled studies to examine the cost-effectiveness of an intervention are still rare in the field of audiology. This paper describes the design of a randomized controlled trial to evaluate the effectiveness and cost-effectiveness of the VEP integrated care programme compared with usual care in reducing the 'need for recovery after work'.

## Methods/Design

### Study design

**Figure 1:**
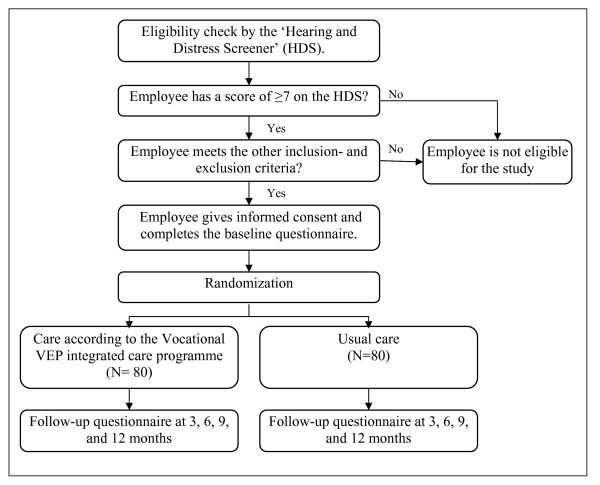
**Study design**.

A two-armed randomized controlled trial, with a follow-up of one year will be performed. Employees randomly allocated to the intervention group will receive the VEP integrated care programme; employees allocated to the control group will receive usual care. Measurements will take place at baseline, and 3, 6, 9, and 12 months after baseline measurement. The study design is presented as a flow diagram in Figure 1. The study design, as well the measurements and the structure of the VEP integrated care programme are described in the following paragraphs. The study protocol is approved by the Medical Ethics Committee of the VU University Medical Center. Participation is voluntary and all participants will sign an informed consent prior to inclusion.

### Study population

The study population will be recruited from three occupational health services (OHSs) in The Netherlands. One OHS is attached to a university including a university medical hospital. The second is the OHS of an international steel industry company. The third OHS is attached to an airline company. Employees will be invited to participate during organizational periodical health screenings (PHSs) if their pure-tone audiogram shows a hearing impairment (for a definition of hearing impairment see Inclusion and exclusion criteria for the study population), or participants can present themselves after reading a flyer of the study distributed among the employees of the participating companies. Potential participants will be selected on the basis of both measured hearing impairment and the self-rated level of distress (i.e., experience of problems at work). To screen employees for eligibility on these two factors, the 'Hearing and Distress Screener' (HDS) is developed. The HDS contains five items. Table 1 shows the items and the codes assigned to the answer category per item. Items 1 to 3 are obtained from the distress scale of the Four-Dimensional Symptom Questionnaire (4DSQ) [20,21]. A recent study showed that a high score on those questions can be used as a quick screener for distress [22]. Item 4 specifically addresses the individual's hearing ability at work. Item 5a requests employees to perform the 'National Hearing Test' [23,24]; an adaptive speech-in-noise test that uses digit triplets that are presented against a background of masking noise according to an adaptive procedure. The test measures the speech-reception-threshold (SRT) corresponding to 50% intelligibility. The scores of the 'national hearing test' are classified into three categories representing: good (SRT_n _< -5.5 dB), insufficient (-5.5 ≤ SRT_n _≤ -2.8 dB), and poor hearing (SRT_n _> -2.8 dB) [25]. If the participants have a hearing impairment based on regular pure-tone audiogram (for a definition of hearing impairment see Inclusion and exclusion criteria for the study population), the participant scores also 4 points for item 5 (item 5b). The total score on the HDS can range from 0 to 14. A total score of 7 or more is the criterion cut off for study inclusion. This screening method allows researchers to approach hearing impaired employees who experience problems at work. The HDS will be available over the internet.

**Table 1:** Hearing and Distress Screener (HDS); the items, answer categories, and scores per answer category

	No	Sometimes	Regularly
1. During the past time, did you suffer from listlessness?	0	1	2

2. During the past time, did you suffer from worry?	0	1	2

3. During the past time, did you feel tense?	0	1	2

4. Did you suffer from hearing problems at your workplace?	0	4	4

	Good	Insufficient	Poor

5. a. The score of the National hearing Test?	0	4	4

	No	Yes	

b. Or a mean pure-tone hearing loss at 1, 2, and 4 kHz in at least one ear > 25dB hearing loss.	0	4	

#### Inclusion and exclusion criteria for the study population

Inclusion criteria

1. Diagnosis of hearing impairment (i.e., mean pure-tone hearing loss at 1, 2 and 4 kHZ in at

2. least one ear > 25 dB Hearing loss) or a score of 'insufficient' or 'poor' on the National Hearing test

3. A score of ≥ 7 on the 'Hearing and Distress Screener'

4. Age above 18 year

5. Paid work for at least 8 hours a week

6. Able to complete questionnaires in Dutch

7. Available for the study for the following 12 months

Exclusion criteria

1. Tinnitus is the primary condition affecting the individual

2. Already referred to or received the VEP integrated care programme during the past 12 months

3. Not willing or unable to comply with the VEP integrated care programme

4. Employee is pregnant

Employees with a score of 7 or more on the HDS and who indicate to be willing to participate, will be contacted by the researcher by e-mail or telephone. During this contact, the researcher provides information about the implications of participation in the study and sends written information to the employee's home. The researcher then plans a face-to-face appointment with the employee about one week after having sent the written information. During this appointment the inclusion and exclusion criteria will be checked (Inclusion and exclusion criteria for the study population), informed consent will be obtained and the baseline questionnaire will be completed. At the end of the appointment the employee will be randomized to the intervention or control group.

### Randomization and stratification

All eligible participants will be randomly assigned to the intervention or the control group after the baseline measurements. To prevent an unequal distribution among groups, employees will be pre-stratified on two prognostic factors before randomization. The first one is requirement of hearing protection at the workplace. The second prognostic factor is psychosocial related problems based on the HDS (score of 3 or more on the three distress items (items 1 to 3) of the HDS). This results in a total of four strata. Furthermore, block randomization, with blocks of four, will be applied to ensure equal groups sizes within each stratum and for practical reasons. An independent statistician will prepare the randomization schedule by using computer-generated random numbers. The research assistant will arrange sealed envelopes before the start of the study containing either a referral to the intervention group or to the control group. When the baseline questionnaire is completed, the employee can choose an envelope in the correct stratum that contains the randomization under the researcher's supervision.

### Sample size

'Need for recovery after work' is the primary outcome of this study and was therefore used for the power calculation. The 'need for recovery after work' scale comprises 11 dichotomized items [26]. Based on previous findings [12,27] the expected effect size is 0.40. To detect this effect size (assuming a two-sided significance level: 0.05, power (1-β): 80%) 63 persons per group are needed. Taking into account 20% loss to follow up, the required sample size is 160 persons in total, with 80 participants assigned to the control group and 80 participants to the intervention group.

### Blinding

Due to the nature of the intervention, it will not be possible to blind the participants, the multidisciplinary team of the VEP integrated care programme, or the OPs of the companies to treatment allocation. After randomization, all participants will receive a non traceable research code consisting of consecutive numbers. A research assistant will enter all data into the computer using these research codes. Thus, the researcher who will analyse the data will be blinded.

### Co-intervention and compliance

Co-interventions cannot be avoided. Information about all treatments and co-interventions received by the participants will be collected by means of questionnaires on health care use in both the intervention and the control group. In the intervention group, the compliance with the recommendations and advices of the VEP integrated care programme will be measured by asking employees which intervention components they received. These recommendations and advices will be compared with the delivered recommendations and advices by the professionals.

### Study groups

#### Intervention group

The participants in the intervention group will be referred to the Audiological Clinic of the VU University Medical Center and receive the VEP integrated care programme.

#### Description of the VEP integrated care programme

The VEP integrated care programme comprises an assessment of auditory function, work demands and personal characteristics by a multidisciplinary team comprising an audiologist, OP, social worker, and psychologist. The VEP integrated care programme is extensively described elsewhere [18] and briefly outlined here.

As soon as an employee is assigned to the VEP integrated care programme, a work-related questionnaire is sent to the employee's home. The employee is asked to fill in the questionnaire and bring it to their consultation at the audiological clinic. This questionnaire assesses the type of work, the work environment, and activities, including hearing activities at work [7].

In the clinic, the hearing status of the employee is measured using auditory tests. Next to regular pure-tone and speech audiometry, SRT tests (presented in quiet and against a background of steady state noise and fluctuating noise) [28,29] are performed. These SRT test aim to measure a person's ability to understand speech in noise by estimating the signal-to-noise ratio (SNR) at which the participant reproduces 50% of the sentences correct. A spatial speech in noise test with interfering speaker is performed to examine hearing in an ecologically valid setting [30]. Furthermore, a loudness scaling test measuring the ability to identify sounds, and a test for localisation [31] are added if indicated.

**Figure 2:**
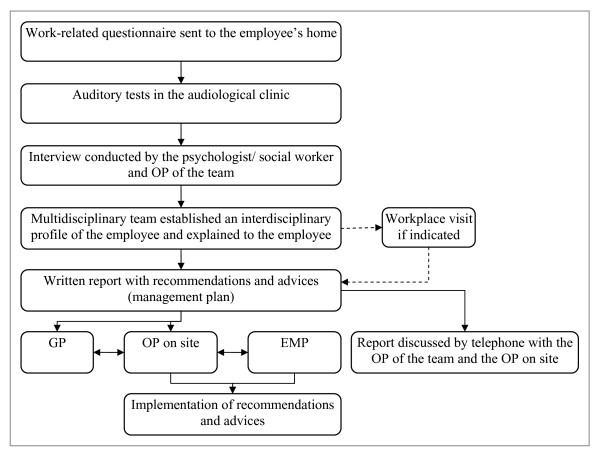
**Schematic overview of the VEP integrated care programme**.

During a semi-structured interview performed by the psychologist or social worker, the psychosocial status of the employee, and his/her specific needs, attitudes towards hearing loss, work and work-related problems are evaluated. This interview is also attended by the OP of the team to evaluate the (person's perspective on) work-related problems. After the tests and interview, the results are examined and considered by the multidisciplinary team consisting of the psychologist or social worker, the OP, and the audiologist in order to establish an interdisciplinary profile of audiological, personal and occupational characteristics. In a multidisciplinary team meeting, all findings are discussed. A written report is then compiled. This report includes recommendations for the employee and covers a proposed management plan which is discussed by telephone with the OP on site. The request of the OP on site is to supervise the implementation of the recommendations and advices. The participant's general practitioner receives a copy of the written report. Finally, the results and conclusion are discussed with the employee. Possibilities of technical, speech-therapeutic and/or psychosocial interventions are then considered. If indicated, the workplace itself is visited in order to inspect acoustical and occupational circumstances. A schematic figure of the VEP integrated care programme is presented in Figure 2.

#### Control group

Participants in the control group will receive usual care, based on advices of their own OP. The OP can refer the employee to the general practitioner, ENT specialist, or to an audiological clinic for further audiological diagnostics and care for hearing impaired persons with work-related difficulties. Furthermore, participant with noise-induced hearing loss will receive care based on existing guidance [16]. Treatment is not restricted in any way but is accurately registered using questionnaires.

### Data collection

Outcome measures will be collected either by online questionnaire or via paper-and-pencil copies. The hard copy questionnaires, including reply paid envelopes, will be sent by regular post to participants without access to or who do not use Internet. Data will be collected at baseline and after 3, 6, 9, and 12 months after baseline measurement. To reduce loss to follow-up, reminders will be sent by e-mail or regular post (paper-and-pencil users) to ask participants to complete the questionnaires with a maximum of three reminders. If they do not wish to further participate in the study, the reasons for their withdrawal will be recorded.

### Outcome measures

#### Primary outcome measure

##### 'Need for recovery after work'

Primary outcome is defined as the 'Need for Recovery (NFR) after work' [26]. This is the degree to which employees are able to recover from short term effects of fatigue and distress caused by work activities. The 'NFR after work' scale is a subscale of the Dutch questionnaire on the experience and assessment of work (VBBA) and comprises 11 dichotomous items (yes/no). The scores on the items are summed and transformed into a scale ranging from 0 to 100, with a higher score indicating a higher 'Need For Recovery after work'. The 'NFR after work' scale is a valid and reliable (Cronbach's alpha; 0.88) instrument for measuring 'need for recovery after work' in The Netherlands [26,27,32].

#### Secondary outcome measures

##### Coping with hearing impairment

The Communication Profile for the Hearing Impaired (CPHI) [33,34] assesses whether a person's communication is effective and whether adequate hearing strategies are used to cope with hearing impairment in daily life. The CPHI reflects an individual's coping-with-hearing-impairment behaviour. The short form of the CPHI with six subscales containing 35 items [33,34] will be used in this study. The six subscales are; 'maladaptive behaviour', 'verbal strategies', 'non-verbal strategies', 'self-acceptance', 'acceptance of loss', and 'stress and withdrawal'. Items are answered on a five-point scale (score 1-5), with either a frequency continuum or an agree- disagree continuum. The item scores of the scales will be recoded such that low scores indicate potential problems. The CPHI is a reliable and valid instrument for application in The Netherlands [33,34].

##### Distress

Distress is measured by the distress scale of the 4DSQ [21]. This scale contains 16 items. The items are scored on a five-point scale using the response categories: 'no', 'sometimes', 'regularly', 'often', and 'very often or constantly'. Every symptom will be recoded into 0 points ('no': symptom is absent); 1 point ('sometimes': doubtfully present); or 2 points ('regularly/often/very often': present at a clinically significant level). Thus, the range of the summed scores is 0-32. Summed scores higher than 10 indicate moderately elevated distress; score higher than 20 indicate strongly elevated distress [21]. The 4DSQ is a reliable and valid instrument for application in The Netherlands [35,36].

##### Health related quality of life

The EuroQol EQ-5D [37] will be used to assess the employees' health related quality of life. The questionnaire describes the respondent's general health status on five domains: 'mobility', 'self-care', 'usual activities', 'pain/discomfort' and 'anxiety/depression', each with three levels (no problems, some problems, and extreme problems). Utilities for the health status will be estimated using the Dutch EQ-5D tariff [38]. Quality-adjusted life years (QALYs) will be calculated by multiplying the utility of a health state with the time spent in this health state. Transitions between health states will be linearly interpolated. Also, the patient's own valuation of his health status will be measured using a Visual Analogue Scale (VAS).

##### Self-efficacy

The General Self-Efficacy Scale (GSES) [39] measures the employees' general beliefs of self-efficacy. Self-efficacy is defined as "the belief of a person in his/her ability to organize and execute behaviours necessary to produce attainments" [39]. It includes 12 items with a five-point response scale from 1 (I totally agree) to 5 (I totally disagree) with sum scores ranging from 12 (most negative) to 60 (most positive) [39]. It is a reliable and valid instrument for use in The Netherlands [39,40].

##### Behavioural determinants

The behavioural determinants attitude, social influence and self-efficacy (ASE) can provide insight into (the intention to perform) the desired behaviour. Questions about attitude, social influence, and self-efficacy for hearing impaired employees are formulated based on questions often used in health promotion research [36,41]. Six questions are included using a five-point response scale from 1 (I totally agree) to 5 (I totally disagree). Sum scores for each behavioural determinant range from 2 (most negative) to 10 (most positive).

##### Sick leave and work productivity

Self reported sick leave days are determined over the past three months using the PROductivity and DISease Questionnaire (PRODISQ). The questions cover relevant aspects of sick leave including compensation mechanisms that may reduce productivity losses at work [42]. Additional data on sick leave and diagnosis are collected from the records of the OHS and Human Resource department of the participating companies after one year follow up to compare the self reported sick leave with the registered sick leave. Self-reported work productivity (i.e., presenteeism) is measured using an item from the WHO health productivity questionnaire [43,44] which asks participants to report their overall work productivity on a ten-point scale in the past three months.

##### Health care use

Health care use is measured using a modified version Tic-P questionnaire [45]. The number of contacts with health care providers specialised in hearing or audiology and all other relevant health care providers (i.e., primary, secondary, psychosocial, and occupational care), the number of medications used, and the number of diagnostic examinations performed during the past three months are included in this questionnaire.

#### Prognostic outcome measure

Psychosocial workload is considered to be a potentially prognostic factor for the primary outcome measure, 'need for recovery after work'. A validated Dutch version of the Job Content Questionnaire (JCQ) [11] will be used to measure the employee's psychosocial work characteristics. Answers are given on a four-point scale, varying from strongly agree to strongly disagree. Scale scores are computed using the job content instrument scale construction formulae [46]. Four subscales are derived from the JCQ: psychological job demands (5 items with a scale score from 12 to 48), physical job demands (5 items with a scale score from 5 to 20), job control (9 items with a scale score of 24-96), and social support (8 items with a scale score from 8 to 32). A higher score indicates a higher psychological job demand, job control, and social support respectively. It is a reliable and valid instrument for application in The Netherlands [11,36,41].

For an overview of the outcome measures and measurement moments see Table 2.

**Table 2:** Schedule of outcome measurements

	T0	T1	T2	T3	T4
**Primary outcome measure - questionnaire**					

'Need For Recovery after work' - NFR after work scale	**×**	**×**	**×**	**×**	**×**

**Secondary outcome measures - questionnaire**					

Coping with hearing impairment - CPHI	**×**	**×**	**×**		**×**

Distress - 4DSQ	**×**		**×**		**×**

Health related quality of life - EQ-5D/VAS	**×**	**×**	**×**	**×**	**×**

Self-efficacy - GSES	**×**		**×**		**×**

Behavioural determinants - ASE	**×**		**×**		**×**

Sick leave - PRODISQ	**×**	**×**	**×**	**×**	**×**

Work productivity - PRODISQ	**×**	**×**	**×**	**×**	**×**

Health care use - Tic-P	**×**	**×**	**×**	**×**	**×**

**Prognostic outcome measure - questionnaire**					

Psychosocial workload - JCQ	**×**		**×**		**×**

Schedule of outcome measurement at baseline (T0), at 3 months (T1), 6 months (T2), 9 months (T3), and 12 months (T4) after baseline measurement

### Costs

A bottom-up cost price calculation will be performed to calculate costs of the intervention and control group. Total costs consisted f costs for the intervention, costs for productivity loss, costs for sick leave, and health care costs. The intervention costs include costs for the implementation of the intervention (i.e., materials and the consultation of the team for the VEP integrated care programme). Costs for productivity loss are costs due to sick leave and costs due to reduced self-reported work productivity. These costs will be calculated according to the human capital cost approach and the friction cost approach, based on income as provided by the employee or as derived from age- and sex-specific income of the Dutch population [47]. The human capital approach assumes that the productivity losses associated with the whole period of sick leave and reduced work productivity. The basic idea of the friction cost approach is that the amount of production lost due to disease depends on the time-span organizations need to restore the initial production level.

Health care costs include costs of the visits to health care providers, diagnostic examinations, and medication due to health problems (i.e., health care problems due to the hearing impairment, but also due to all other complaints). The health care costs will be evaluated according to the prices suggested in the guidelines for economic evaluation in The Netherlands [48]. If cost-guidelines are not available, costs will be estimated using real prices. Medication costs will be valued using prices of the Royal Dutch Society for Pharmacy (Z-index) [49]. All prices are adjusted for the year of 2011 using consumer price index figures.

### Statistical analyses

All analyses will be performed according to the intention-to-treat principle. Baseline characteristics of employees in the two groups will be compared using descriptive statistics. Linear and logistic regression will be performed. Furthermore, analyses at the level of the OHSs are performed by the use of multilevel analysis. To assess whether protocol deviations cause bias, the results of the intention-to-treat analyses will be compared to per-protocol analyses, from which employees who are not treated according to the procedure of the intervention. For all analyses, a two-tailed significance level of < 0.05 is considered statistically significant. Linear and logistic regression analyses will be performed with SPSS 18.0 (SPSS Inc. Chicago, Illinois, USA). The multilevel statistical analyses are performed with MIWin 2.0.

### Economic evaluation

In the economic evaluation the costs and consequences of the VEP integrated care programme and usual care will be compared. As mentioned in the introduction, hearing impairment has consequences with potentially substantial costs for the companies, as well for the society. Since interventions in occupational care in The Netherlands have to be paid by employers, the economic evaluation will be conducted from both a company and a societal perspective. From the societal perspective, all costs and consequences of the intervention will be taken into account regardless of who pays for them. From the company perspective, only costs due to the intervention, productivity, and sick leave will be taken into account.

Missing data will be imputed using a multiple imputation procedure according to the multivariate imputation by chained equations (MICE) method [50,51]. To calculate incremental cost-effectiveness ratios (ICER), the difference in mean costs between the intervention and control group will be divided by the difference in outcome measures between the two groups. Confidence intervals (95%) will be estimated using bias corrected and accelerated (BCA) bootstrapping [52]. The cost-effectiveness ratios will be graphically presented in a cost-effectiveness (CE) plane [53]. Cost-effectiveness acceptability curves (CEAC) will also be estimated [54].

### Process evaluation

Additional to the effect evaluation and the economic evaluation, the process of the VEP integrated care programme will be evaluated. Two models for process evaluation will be combined, namely the model of Steckler and Linnan [55] and the RE-AIM model [56,57]. In this study we will evaluate the following six components, which are derived from these two models; recruitment (procedures used to recruit employees for this study); context (characteristics of the participants and companies that affect the VEP integrated care programme); reach (number of participants reach for the HDS, participants who are willing to participate in the study and number of participants in the study groups); dose delivered (the amount of advices and recommendations delivered by the team of the VEP integrated care programme); dose received (the extent to which employees use advices and recommendations by the team of the VEP integrated care programme) and perceived effectiveness (participants' and professionals' view of effectiveness of the VEP integrated care programme for the participants). In addition, satisfaction of employees with the VEP integrated care programme is measured using a modified version of the Patient Satisfaction with Occupational Health services Questionnaire (PSQOH) [58]. Questionnaires for the process evaluation are assessed in the intervention group at three and six months after baseline.

## Discussion

Hearing impairment at the workplace has consequences for the hearing impaired individual, the company, and society [4,7]. So far, the majority of studies on the relationship between hearing impairment and work, published in the international literature, are restricted to the causes of noise-induced hearing loss, on how to protect employees from dangerous noise levels, and on the development of hearing protection programmes [18]. The specific problems in communicative function encountered by workers with hearing impairment in the workplace have rarely been addressed in research or in clinical practice protocols. Hétu et al. [9] argued that a multidisciplinary approach is crucial for a successful management of complex problems in the workplace. To the best of our knowledge, this is the first study that describes the evaluation of an integrated care programme for hearing impaired employees that targets both the individual capacities as well as the work environment according to a randomized controlled design.

The VEP integrated care programme is unique because usual care often does not include adequate interdisciplinary diagnostics and audiological rehabilitation procedure other than hearing aid fitting for addressing the problems related to hearing impairment in the workplace. In particular the psychosocial problems are often left behind and protocols for improvement of the workplace by applying accommodations for the hearing impaired employee are not broadly implemented.

Results of this study will be analysed within the context of the ICF classification. From the perspective of the society and employer the study will evaluate the components of the health condition (i.e., hearing impairment at the workplace) such as activities, participation and environmental and personal factors, namely with questionnaires about work demands, 'need for recovery after work', and general health status. In this article the design of a study to investigate the effectiveness, cost-effectiveness, and feasibility of the VEP integrated care programme for hearing impaired employees is described.

### Methodological considerations

A strength of the current study is the randomized design. Another strength is that not only the effects of the VEP integrated care programme will be evaluated, but also the cost-effectiveness and the process of the programme. Finally, in this study presenteeism is measured. This is important, because even if an employee is not absent from work, hearing impairment can cause a reduction of work productivity [14].

Hearing impairment is not a disorder that is visible and it usually develops gradually. Hence, it is often not recognized or considered to be a problem by employees. Many people perceive their hearing impairment as part of themselves and are therefore not alarmed by it. The employees who do recognize the problem of their hearing impairment may not report it, because of shame or the fear for stigma [8]. It may therefore be difficult to measure a difference in effect (i.e., 'need for recovery after work') between the intervention and control group. Another limitation of this study is that it is not possible to blind the employees and multidisciplinary team to the intervention. Selection bias is possible because of self selection of companies participating in this type of studies. It may be expected that only companies in which hearing impairment is considered as an important health condition, for example because of knowledge about risk of noise, will participate in this study. It may also be argued that only companies that recognise the importance of preventive interventions participate. For two of the participating companies (airline and steel industry), this may have influenced their decision to participate. However, for the university, hearing protection is less of an issue. Hence, it is argued that the participating companies represent a good mix of employers.

### Policy implications

This VEP integrated care programme is a promising way to help reducing work disability and the burden for hearing impaired employees. If this study will prove its effectiveness, the VEP integrated care programme can foster the maintenance, and improvement in the well-being of employees with hearing impairment by facilitating and retention of their work through the VEP broadly. When proven cost-effective from the company perspective, it can be considered as a useful intervention in other occupational health care settings to prevent disabilities for hearing impaired employees. Furthermore, if cost-effective from the societal perspective, it will be worth considering inclusion of the VEP integrated programme in the basic package of social insurances. It is expected that the results of this study will become available in 2014.

## Abbreviations

ASE: attitude, social influence and self-efficacy; BCA: bias corrected and accelerated bootstrapping; CE: cost-effectiveness; CEAC: cost-effectiveness acceptability curves; CPHI: communication profile for the hearing impaired questionnaire; dB: decibel; dB SNR: decibel signal-to-noise ratio; EMP: employer; ENT: ear nose and throat; GP: general practitioner; GSES: general self-efficacy scale; HDS: hearing and distress screener; ICER: incremental cost-effectiveness ratio; ICF: international classification of functioning: disability and health; JCQ: job content questionnaire; JDC: job demand control; MICE: multivariate imputation by chained equations method; NFR: need for recovery; NVAB: Dutch board for occupational medicine; OHS: occupational health services; OP: occupational physician; PHS: periodical health screening; PRODISQ: productivity and disease questionnaire; PSQOH: patient satisfaction with occupational health services questionnaire; SNR: signal to noise ratio; SRT: speech reception threshold; VAS: visual analogue scale; VEP: vocational enablement protocol; WHO: World Health Organization; 4DSQ: four dimensional symptom questionnaire.

## Competing interests

SEK and STG are each involved both as researcher and as clinician in this study. They are the psychologist and the audiologist of the multidisciplinary team of the VEP integrated care programme respectively.

## Authors' contributions

AHMG is responsible for the data collection and drafted the manuscript. SEK and JRA are the guarantors of the study. SEK and STG designed the intervention programme. AHMG, SEK and JRA developed the study design. JRA is responsible for the recruitment of companies participating in the study. STG is responsible for the VEP integrated care programme in the clinic. SEK, JRA, JB, STG and JMF gave critical comments on the draft manuscript and had a role in supervision. All authors participated in discussing the design of the study and read and approved the final manuscript.

## Pre-publication history

The pre-publication history for this paper can be accessed here:

http://www.biomedcentral.com/1471-2458/12/151/prepub
